# COVID-19 risk perception framework of the public: an infodemic tool for future pandemics and epidemics

**DOI:** 10.1186/s12889-022-14563-1

**Published:** 2022-11-18

**Authors:** Anna-Leena Lohiniva, Annika Pensola, Suvi Hyökki, Jonas Sivelä, Tuukka Tammi

**Affiliations:** The Finnish Institute for Health and Welfare, Helsinki, Finland

**Keywords:** Risk perception, Risk communication, Infodemic

## Abstract

Understanding the risk perceptions of the public is central for risk communications and infodemic management during emergency and preparedness planning as people’s behavior depends on how they perceive the related risks. This qualitative study aimed to identify and describe factors related to COVID-19 risk perceptions of the public in Finland and to make this information readily available to those who communicate with the public during crises. The study is part of a larger project exploring crisis narratives through a mixed-methods approach. The study was based on a dataset of over 10,000 comments on the Facebook and Twitter posts of the Finnish Institute of Health and Welfare (THL) between March-May 2021. The data were analyzed qualitatively using thematic analysis. The study identified concepts linked with the pandemic risk perception that included knowledge, perceptions, personal experiences, trust, attitudes, and cultural values. The findings resulted in a framework of risk perceptions that can be used as taxonomy and a set of key concepts and keywords in social listening to monitor risk perception during future epidemics and pandemics.

**Supplementary Information:**

The online version contains supplementary material available at 10.1186/s12889-022-14563-1.

## Introduction

Understanding risk perception of the public is critical for risk communicators during a crisis. Risk perception means a subjective assessment of the actual or potential threat to one’s life or, more broadly, to one’s psychological well-being, which often predicts the willingness of the individual to follow public health measures and accept public health messages. Accordingly, how people perceive risk is not necessarily correlated with biomedical risks [1–4]. When the perceived risk is too high, it may lead to panic or denial of the threat; if the perceived risk is low, it may demotivate adherence to public health measures [5]. This is evident from studies conducted during previous epidemics such as the 2003 SARS outbreak, which indicated that perceived higher risk of SARS infection was associated with engagement in more precautionary behaviors, and compliance with infection control policies [6, 7]. Risk perception is a complex phenomenon influenced by multiple psychological, societal, and cultural factors that change in time and place [3, 8, 9]. Recent studies of subjective risk perception towards COVID-19 conclude that risk perception is driven by various factors including demographic factors, individual factors, geographical factors, timing, and cultural factors highlighting the importance of gaining a greater understanding of individual and cultural factors in each context [4, 10, 11].

Risk perception can be monitored by social listening, which means a continuous and systematic process to collect online and offline data about public perceptions and information voids using standard tools. Social listening provides valuable data on public opinions that, through conversations with the public, can shed light on the dynamics of the pandemic. For example, one can detect misinformation, explore public sentiments, measure public adherence to prevention measures, and understand reasons for noncompliance with the measures as well as understand risk perceptions [12–14]. Social listening can take many forms. Some social listening processes are based on big data [15–18]. Often dashboards are used to present the findings, such as the Red Crescent COVID-19 dashboards that were piloted in selected countries during the pandemic and the WHO Early Artificial Intelligence-Supported Response with social listening that monitors COVID-19-related online discussions in 30 countries [19, 20]. Some projects have focused on smaller datasets based on manual browser searches and qualitative methods [21, 22]. Other projects have collected field-based data on rumors such as a real-time rumor-tracking pilot in Côte d’Ivoire that leverages existing public communication structures, including hotlines and community health workers, to submit rumors to a central database for rapid coding and visualization of the findings on dashboards [23].

Social listening is a critical part of infodemic management, which is defined as the management of an overload of information including misinformation, rumors, and risk perceptions. Infodemics can severely undermine the public health measures of authorities. Information overload is known to affect risk perception and create information anxiety that can develop into information avoidance [12, 13]. It is therefore important that as part of infodemic management public health authorities and risk communicators understand what is driving the risk perception. This allows risk communicators to inform the public about risks, influence behavioral change, and encourage participation in decision-making about emergency measures.

To advance infodemic management in Finland, the Finnish Institute of Health and Welfare (THL) developed a pandemic-related risk perception framework that serves as a taxonomy to be used in social listening to monitor the risk perception of the public during future pandemics and epidemics. The study is part of a larger project exploring crisis narratives through a mixed-methods approach using qualitative research and social media analytics that include developing a platform of essential information for future crisis preparedness and planning in Finland in which the taxonomy can be embedded. This paper describes the findings of a qualitative study that describes concepts linked with the pandemic-related COVID-19 risk perceptions of the public in Finland to inform the risk perception framework.

## Methodology

This is a qualitative study based on public comments on the Facebook and Twitter posts of the Finnish Institute of Health and Welfare (THL) from March 1, 2021 - May 31, 2021. THL is a public health institution in Finland that provides guidance and recommendations to the ministries in the national pandemic response. Likewise, THL is a pandemic-related information source for the public in Finland.

During the time of the data collection, there was both a rise of the pandemic with the gradual strengthening of the public health measures as well the decline of the pandemic with the easing of the measures that accordingly gather a rich variety of risk-related perceptions. On the second of March 2021, the number of new confirmed COVID-19 cases in the country was 756 which gradually reached almost 900 new cases by mid-March 2021. From April 2021 the caseload started to decrease so that on May 31st of May 2021 there were just 150 new confirmed cases in the country.

The data was retrieved by using Emplifi, a social media management tool that is used in THL’s daily social media communication by the social media managers. All published posts are given a tag for managing and reporting purposes by the social media manager, and this allowed the retrieval of all posts tagged with “corona”, indicating COVID-19 as the main theme of the post. These posts and accompanying comments were saved to an Excel file for further inspection. The specific subject matter of the COVID-19-related posts ranged from weekly updates on the pandemic situation, to new related studies, and to THL recommendations on topics such as mask use and remote working practices. The comments of an individual post were collected 3 days after its publication at the earliest. The use of a social media tool ensured the systematic retrieval of each relevant post. From March-May 2021, THL made 367 Facebook and 546 Twitter posts, of which 214 and 316 were corona-tagged respectively. According to statistics retrieved from Emplifi, about 80% of the THL Facebook audience are female, and 50% of the audience are 35-54-year-old women. In contrast to Facebook comments, Twitter comments frequently included trolling; online bullying by deliberately trying to offend, cause trouble or attack others on social media.

The data was processed according to THL ethical guidelines. Anonymization was carried out manually by deleting any personal names or specific locations which referred directly to the comment authors. Locations indicating general areas (e.g., capital region, Southern Finland) as well as mentions of entire cities and countries were preserved. Likewise, personal names referring to people in the public eye (e.g., decision-makers, and local healthcare experts in the pandemic context) were left intact when they appeared in the content of the comments. All author information related to individual comments was erased. Comments in languages other than Finnish, and comments that were not related to the pandemic context (e.g., spam), as well as comments without verbal input (e.g., emojis, links without accompanying text), were discarded.

The final dataset consisted of 144 Facebook and 123 Twitter posts. The posts collected from Facebook had 9792 comments and 2612 unique authors, while the Twitter posts had 932 replies and 420 unique authors. THL’s replies to comments and questions varied among posts and platforms but were included in the data in order to preserve the context of the discussions in the comment section. Although the number of posts from both platforms is relatively equal, the tendency of a Facebook post to elicit interaction among followers was significantly higher. However, it should be noted that it was only possible to include Twitter replies in the dataset as retweets are not available in Emplifi.

The data analysis was based on thematic analysis [24] by two researchers using NVIVO software to code and categorize the data and a MIRO online board to synthesize and interpret the findings. The two researchers began the process by familiarizing themselves with the dataset as a whole by reading through the chains of comments followed by coding words, sentences, or paragraphs that reflected the scope or extent of the pandemic. They independently coded different data sets but coordinated regularly to review each other’s coding and to determine the final set of codes in consensus. They individually identified categories, followed by a number of joint reviews, to determine the final set of themes that describe concepts that are linked to risk perception. See Supplementary material 1 for a code book.

### Findings

**Fig. 1:**
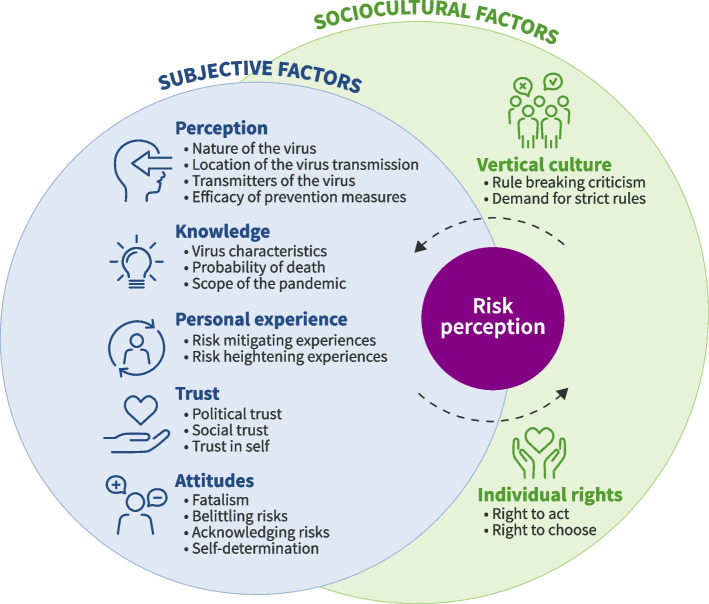
Pandemic risk perception framework

The concepts associated with risk perception resulted in a taxonomy of eight themes that included five subjective factors: knowledge, perceptions, personal experience, trust, and attitudes, as well as two cultural factors that included respecting the rights of individuals and vertical culture. The themes were divided into 21 sub-themes See Fig. 1.

### Knowledge

Knowledge was defined as information about biomedical factors about the virus and the pandemic. Sub-categories that were identified as subjects that influence risk perception included virus characteristics, numerical and statistically relevant probability of death, and the scope of the pandemic. Virus characteristics that increased risk perception included the strength of the virus associated with the severity of the symptoms, speed of the transmission, contact time, as well as modes of transmission associated with the airborne transmission, in addition to changes in the virus associated with virus mutations, asymptomatic nature of the virus and unknown nature of the virus. The probability of death was conceptualized based on the number of people dying and the types of people dying. The risk was perceived as more severe when those who were dying were young people.

*“This is nothing, but a normal spring only made to look like a pandemic through vast testing. Look at the real numbers! Only the number of deaths and hospitalizations matters – which are not bad at the moment.”*

### Perceptions

Perception was defined as an interpretation, comprehension, and understanding of risk-related concepts. They included the nature of the virus, the location of the virus transmission, the transmitters of the virus, as well as the efficacy of prevention measures.

The perceptions of the virus as contagious or aggressive were recognized to increase risk perception. The risk of virus transmission was localized into environments that included small, narrow, crowded places, indoor places, and outside of Finland. The transmission was also localized into certain types of people who were perceived as the transmitters of the virus, such as careless people, people from different cultural and ethnic backgrounds, those who were younger, people who do not follow prevention measures, as well as people with a social lifestyle that includes parties, events, and travel. The transmitters of the virus also included people who could not avoid social contact due to their professions such as healthcare workers, people working in the service sector, as well as those who had an asymptomatic infection.

*“Smart people would postpone their graduation parties to the end of the summer. If we mess up the good progress the whole summer will be ruined.”*

If infection prevention measures such as social distancing or mask use were perceived as inefficient in the prevention of the pandemic, worry about the pandemic increased. These measures were seen as inefficient as the virus was considered stronger than the measures and virus transmission was therefore seen as inevitable. The prevention measures were also deemed inefficient because they were seen as illogical, such as restricting restaurant hours, or as something impossible to implement in everyday life such as distancing from other people while on public transport or shopping. Lastly, the measures were also perceived as ineffective based on real-life observation during which people who had taken measures contracted COVID-19, which heightened the risk perception. On the contrary, protecting oneself and others from COVID-19 by adopting infection prevention measures reduced risk perception.

*“If desert sand can fly all the way from Sahara to Finland, then why do some people think that a flimsy mask or a two-meter distance would stop viruses from spreading.”*

*“I will keep on going, as usual, using facemasks, distancing, taking care of hand hygiene, and limiting my activities to essential ones. I’ll keep on acting like this as long as it is necessary.”*

### Personal experiences

Accounts of personal experiences with COVID-19 or experiences of friends or family that included pain, suffering, death, prolonged symptoms, financial problems, or social challenges increased risk perceptions. Personal accounts of COVID-19 with mild symptoms, symptoms comparable with seasonal flu, and short duration as well as the absence of perceptible changes to one’s social and financial situation reduced risk perception.

*“I’ve had corona and I know that even a mild case is not easy.”*

*“I know many people who’ve had corona, even risk group people, and they’ve all had a mild case.”*

### Trust

Discussions that reflected reduced trust towards political entities and media included comments on unreliable reporting or scandal-seeking reporting, arbitrary or rapidly changing infection prevention recommendations, and lack of action by the authorities to prevent the transmission of the virus in Finland. Discussions that reflected reduced trust towards other people (societal trust) manifested in comments that criticized others for not following the infection prevention measures. Mistrust in personal capabilities to self-protect against COVID-19 is reflected in heightened risk perception of the pandemic, while trust in one’s ability to survive a possible infection decreased the perceived risk.

*“Policymakers are making decisions with votes in mind, at the expense of people’s health.”*

*“If I had the choice, I’d get myself corona straight away and be done with it. All people I know have had a mild case; my immune system could handle it.”*

### Attitudes

Attitude was defined as a relatively stable feeling or way of thinking that affects a person’s behavior. Attitudes that mirrored risk perceptions included belittling and acknowledgment of the pandemic as well as the importance of self-determination and fatalism. Belittling included statements about the risks of the pandemic as overexaggerated, such as coronavirus being nothing more than seasonal flu. Such statements reflected reduced risk perceptions. Whereas statements indicating acknowledgment of the pandemic as a real and serious threat with various health, social and financial implications, reflected heightened risk perceptions. Comments in which individuals highlighted concerns about a loss of personal agency in their life as a result of infection prevention and control restrictions mirrored increased risk perceptions towards the pandemic. Finally, fatalism was evident from comments in which respondents emphasized pandemics as a natural order of the universe, and actions of an individual were not seen as having an effect in the course of the pandemic. The discussions aimed to normalize the pandemic and pandemic-related risk perception.

*“Corona is a bunch of hogwash. I’ve kept living my life as usual and seeing my friends. When restaurants open again, I’m certainly going.”*

*“Some people see corona as a joke and downplay its severity. Everyone should read about the corona experiences of those infected with strange and long-term symptoms.”*

### Cultural factors

Discussions also referred to broader sociocultural factors including the rights of individuals to know, to be informed, or to have the freedom to take any actions as a principle that everyone was entitled to but that the pandemic was threatening to take away, which heightened risk perception towards the pandemic. In addition, comments reflecting vertical culture, in which people focus on complying with authorities, express willingness to comply with the strict rule, and request more and stricter infection prevention measures, were perceived as reducing risk perceptions related to the pandemic.

*“It’s true we should stay home while sick. Other than that, we have the right to live and be active without a mask [..].”*

*“People are frightened and stalk each other’s actions. It’s just unhealthy the way people are acting. I wish this madness and fuss would come to an end.”*

## Discussion

This study provided valuable insights into the pandemic-related risk perception of the members of the public in Finland by identifying and describing related concepts including knowledge, perceptions, experiences, trust, and sociocultural values. Although the concepts require further validation to be generalized to the public at large, saturated qualitative data is commonly used to guide evidence-based risk communication efforts [25, 26]. Risk communicators can monitor risk perception by following the public discussion around risk perception-related concepts and accordingly intervene with the right type of information at the right time.

The study showed that lack of knowledge increases pandemic-related risk perceptions, as identified in related studies that have found a positive relationship between disease-related knowledge and perceived risks [27–30]. To reduce risk perceptions, risk communicators need to capture information voids in real-time that allow them to focus on delivering fact-based high-quality information to the public, which subsequently reduces the perception of risk. The study showed that the need for knowledge was linked with virus characteristics, the scope of the pandemic, and the evidence-based probability of death, which indicated the need to communicate numerical data, which is often considered the information of experts. Some studies show that non-experts can also have a good understanding of numbers, probabilities, and their conceptualization [31], whereas other studies support the notion that people often have difficulty understanding numerical risks and benefits in health information. This highlights the importance of testing how numerical information is communicated and displayed verbally and visually to the public, and whether it affects risk perceptions and behaviors [32]. In addition, reliable information sources are the basis to acquire credible knowledge and build social trust. False or misleading information may lead to exaggerated fears or a lack of attention to an emerging threat [33].

The study also demonstrated that the pandemic risk perception is linked with various perceptions that can be addressed in when aiming to reduce the fear surrounding COVID-19. For example, the perceptions of the virus as aggressive, unpredictable, or unknown can easily develop into information and rumors that should be addressed. Debunking as a technique to misspell misinformation has become an increasingly popular tactic. Debunking is a special technique to deliver information, in which people are exposed to misinformation but also to facts and confirmatory facts [34]. To be effective, debunking should include a compelling narrative as information alone is unlikely to change inaccurate perceptions [35]. Likewise, prebunking could be used to prevent misinformation generation by inoculating against it. Prebunking draws from the notion that by warning people in advance about possible misinformation, the effect of misinformation is weakened [36]. Both debunking and prebunking have been used effectively during the pandemic in the WHO “Stop the Spread” campaign and the United Nations ‘Verified’ initiative in collaboration with the UK government [37, 38]. Risk communicators develop and disseminate targeted debunking messages, or alternatively to run prebunking campaigns for wider audiences, which subsequently intervenes with the risk perception.

Of all the concepts linked with risk perception, trust is perhaps the most pivotal. Mistrust can easily generate a great deal of fear and anger, particularly when people feel their concerns have been mishandled, they have been misled about the risks, or have been exposed to risks without their consent. Studies about trust suggest that those who display a higher level of trust in a decision perceive less risk than those who trust less [39]. This corresponds with the findings of this study in which discussions that reflected mistrust towards the authorities and media, in the community and self, reflected heightened risk perception. Mistrust evolves in time and place. For example, trust in institutions and authorities have been shown to change during the pandemic corresponding with the adoption of public health measures [40]. Trust has also a context-specific cognitive dimension, meaning that people have beliefs and make decisions about whom to trust and when. For example, the specific actions that people believe contribute to trust in the context of authorities are different from those in the community or in the context of personal relationships [41]. Accordingly, the same trust-building strategies do not apply to both. Trust-building efforts can take various approaches including different interventions. However, it is important to remember that concepts linked with trust and mistrust are context-specific [42]. Successful trust-building efforts have used transparency, including accessibility to information, provision of emotional support, and information on skills and resources as some of the key approaches to building trust [43]. Other successful trust-building approaches include the use of a positive deviance strategy that relies on positive modeling and examples [44] or active listening as well as strengthening the verbal and nonverbal communication skills of health authorities [45].

The study showed that negative personal experiences increased risk perception whereas positive experiences decreased it, which is in line with other studies that show that personal experiences affect risk perception as well as behavior such as vaccine uptake [46]. Personal experience often reflects emotions which makes them powerful tools for communicating risks [47]. Risk communicators can build on the existing stories and use them to decrease or increase risk perception.

The findings of this study indicate that individual risk perception is not only linked with individual factors but also with broader sociocultural values, such as vertical culture and individual rights. Interestingly vertical culture was also identified in a study in China that concludes people in China being influenced by the egalitarian and hierarchical culture that increases their risk perception towards environmental threats [48]. Cross-cultural studies in crisis situations point out that cultural factors may have a greater influence on risk perception than social exposure [49]. It is therefore important that trust-building interventions are culturally competent, starting by valuing diversity [50]. Risk communicators must ensure that communication and messages are aligned with the cultural concepts by reflecting on them during the piloting of the messages. A checklist can be developed for those purposes.

One of the strengths of the study is that the contexts during which the data was collected included a new pandemic wave, during which public health measures were increasingly put in place, as well as the decline of the pandemic, during which public health measures were eased, thereby providing different contextual settings for risk perceptions. In March 2021, the country experienced a new wave of infections caused by the SARS-CoV-2 virus that led to the closing of many public places, events, and sports facilities, home-based work when possible, and limiting the opening hours of restaurants and bars. In April 2021, infections caused by the SARS-CoV-2 virus started to decline and the government implemented regional-based restrictions that allowed areas with fewer cases of COVID-19 to ease public health measures in place. By the end of May, the number of new infections had slowed down and the government lifted most of its restrictions involving social distancing. Accordingly, the study gathered risk-related concepts during a time when risk perceptions were expected to rise and decline. Another strength of the study was the deep and insightful interactions with the data by the researchers that included multiple layers of reflections to determine the final set of concepts. In addition, researchers used a mix of tools such as digital analysis software and online platform to help in the visualization and conceptualization process to support the analysis, which has been shown to be particularly valid methods of theory development and insights as software do not fully scaffold the analysis process [51].

The study also had limitations. Social media sites as a data source may create bias as not all members of society communicate on social media. From the limited background information available, it was evident that the majority of those commenting on the Facebook site were adult females. It was not possible to get any background information on Twitter users. It is also likely that such social media sites gather people who are, on average, more interested in the pandemic and health issues than those who do not communicate via social media. In general, people who are in contact with health authorities during an emergency are often highly emotional and have strong opinions [52] that may add to the bias. It is also not always possible to recognize bots and trolling from authentic comments. Accordingly, it is important to acknowledge that the study may have missed some risk perception-related concepts and further verification is required to be able to generalize the findings to the public. It is also important to acknowledge the reflectivity that is characteristic of qualitative analysis, which may create bias. Social media-based qualitative analysis has also weaknesses. The data are typically short sentences, which makes interpretation of the data in some instances difficult or impossible. Therefore, the links of the concepts to perceptions, attitudes, knowledge, and trust were debated lengthily during the analysis by the researchers. Links between the concepts could not be established at all. Likewise, there can be bias as social media-based data does not provide an opportunity to probe and ask additional questions from those posting as during face-to-face data collection. Therefore, future activities of the project include deepening the understanding of the risk perception concepts through focus group discussion. In addition, future activities include validating the concepts through big data to ensure they sufficiently capture the various dimensions of the pandemic-related risk perception and can be embedded in a digital platform where they can serve as keywords for risk perception monitoring.

## Conclusions

The concepts and associated sub-concepts that make up the risk perception framework can be used as search terms to monitor public risk perception during future pandemics and epidemics. The framework will be particularly beneficial for risk communicators and other public health officials who can utilize the framework to formulate effective messages and other risk communication content at the right time during future pandemics and epidemics.

## Supplementary Information

**Additional file 1: Supplementary material 1.** The pandemic codebook of the public risk perception framework.

## Data Availability

Data analyzed during this study are included in this published article.
