# Supplementary material for: COVID-19 risk perception framework of the public: an infodemic tool for future pandemics and epidemics
Source: BMC Public Health. 2022 Nov 18;22:2124. doi: 10.1186/s12889-022-14563-1 (PMC9675166; doi:10.1186/s12889-022-14563-1)
Supplement: Supplementary file 1 — Additional file 1: Supplementary material 1. The pandemic codebook of the public risk perception framework. [file 12889_2022_14563_MOESM1_ESM.docx]

Supplementary material 1. The pandemic codebook of the public risk perception framework

| Theme | Sub-themes | Categories | Examples of codes |
| --- | --- | --- | --- |
| Knowledge | Virus characteristics | Strength of the virus  Modes of transmission  Changes in the virus | The virus develops severe symptoms, speed of the transmission of the virus, short contact time for the transmission, and airborne transmission  Virus mutations, new strains, asymptomatic nature of the virus, changing characteristics of the virus (lack of knowledge about new variants) |
|  | The probability of death | Number of people dying  Type of people dying | Number of deaths in Finland, number of deaths globally  Young people, the elderly, those with underlying health conditions |
|  | The scope of the pandemic | Increasing number of new coronavirus cases | Transmission rate  Number of new cases  Prevalence |
| Perceptions | Nature of the virus | Contagiousness of the virus  Lethal outcome  Comparable with other mild infections | The virus is contagious, the virus is aggressive, deadly; comparable with influenza |
|  | Location of virus transmission | Small, narrow, or tight places  Indoor places  Crowded places  Abroad, outside of Finland | Small streets, elevators, shops, corridors, schools, workplaces, public transport, sports halls, sports activities, events, restaurants, bars etc. |
|  | Transmitters of the virus | People with a careless attitude  People with a social lifestyle | Foreigners, younger people, people who socialize during quarantine and isolation, and others who do not follow infection prevention measures  Parties, events, traveling |
|  |  | People with a profession that necessitates contact with others | HCWs, caretakers, service branch, schoolchildren, students, working age people, people crossing borders etc. |
|  |  | People with asymptomatic infections | No signs or symptoms |
|  | Efficacy/ inefficacy of the prevention measures | Inevitability of the virus transmission  Illogical prevention measures  Real life challenges  Personal observations during the pandemic | The virus is stronger than any measures; the virus will transmit no matter what.  The bar closes after 8 pm, as if the virus only transmits after 8 pm.  We cannot prevent transmission on a crowded bus.  I know people who have taken all measures, yet they have fallen ill. |
|  | Self- efficacy/lack of self-efficacy | Inability to protect self  Ability to protect self | I do not have the opportunity, skills, knowledge, equipment to protect myself.  It is easy to protect self and others |
| Personal experiences | Risk-mitigating experiences | Positive experiences | Mild case, easy recovery, short duration, no other type of repercussions |
|  | Risk-heightening experiences | Negative experiences | Pain, suffering, death, financial issues, social challenges |
| Trust | Political trust/mistrust |  |  |
|  | Societal trust /mistrust |  |  |
|  | Individual trust /mistrust |  |  |
| Attitudes | Belittling | Virus threat is overexaggerated  Pandemic is not a serious problem  Pandemic is comparable to mild infections | The virus is only mild, the virus is not widely spread or not spreading fast.  Corona is nothing more than the seasonal flu. |
|  | Acknowledging | The virus is a real threat | The virus is strong, the virus is widely spread and spreading fast. |
|  | Self-determination | Lack of control in own life | My decision, my life, my responsibility, my choice |
|  | Fatalism | We must accept the pandemic as it is | Virus transmission is normal  Pandemic is part of the natural order  There have been pandemics and there will be pandemics in the future |
| Culture | Individual rights |  | Right to know  Right to be informed  Right to take an action  Right not to comply with infection prevention measures |
|  | Vertical culture | Strict application of norms  Rule-breaking criticism  Enforcement of strict laws and regulations, restrictions, measures | Everyone needs to follow infection prevention measures.  Infection prevention measures must be followed at all times and everywhere.    No exceptions for infection prevention measures.  Those who don’t follow infection prevention measures are selfish, careless, and responsible for the increased number of corona cases.  Laws instead of recommendations.  Recommendations are not enough. |
